# Fault Slip Rates and Seismic Moment Deficits on Major Faults in Ordos Constrained by GPS Observation

**DOI:** 10.1038/s41598-018-34586-2

**Published:** 2018-11-01

**Authors:** Yilei Huang, Qingliang Wang, Ming Hao, Shiyong Zhou

**Affiliations:** 1 Institute of Theoretical and Applied Geophysics, Peking University, Beijing, 100871 China; 2 Second Monitoring Center, China Earthquake Administration, Xi’an, 710043 China

## Abstract

The Ordos Block, surrounded by numerous active faults, is a relatively rigid but dangerous area with many strong historical earthquakes. We derive the block rotation velocity and fault slip rates in this area by using GPS data recorded from 1999 to 2007 and implementing an elastic block model. Instead of assuming vertical faults, as did most previous studies in and around Ordos, we use an improved method to invert for the fault dip angles and construct a closed 3-D fault system in our inversion. The predicted slip rates range from <1 mm/yr to ~ 10 mm/yr. Our results are roughly consistent with geological and other geodetic observations. Using the estimated slip rates, we also calculate the cumulative seismic moment due to fault locking and the released moment from historical earthquake catalogues. A comparison of the two quantities indicates that the Hetao Rift has an unreleased seismic moment equal to a M_w_ 7.9 earthquake, which is also indicated by frequent earthquakes above M6 after 1900.

## Introduction

The Ordos Block (34°N–42°N, 105°E–115°E) lies in the eastern part of the China south-north seismic belt, surrounded by the North China Plain, South China Block and the Tibetan Plateau. Although the interior is rigid and stable, there is an intensive seismic hazard around its periphery. Three M ≥ 8 catastrophic earthquakes, including the deadliest, the Huaxian earthquake, have struck the Ordos Block’s periphery since the 1500s^1^. It is critical to study the kinematic properties of these faults and assess the earthquake hazards in this resource-rich and populous place.

Intensive Global Positioning System (GPS) and other geodetic measurements have been collected in Mainland China since the 1990s. The geodetic observations provide quantitative maps of the surface velocity field within tectonically active regions, shedding light on the spatial deformation and kinematic properties of the crust. There are two end-member approaches to study the crustal deformation. The first one, based on the micro-plate hypothesis, assumes that the majority of the deformation is due to slip on a finite number of major faults forming the boundaries of tectonic micro-plates^2–9^. The other approach assumes that the crust deforms in a continuum manner, as deformation is diffusely distributed across the active plate boundaries^10,11^. The micro-plate model approaches the continuum model when the block size decreases and the number of faults increases, resulting in more continuous deformation^12^.

In this paper, we first do a theoretical forward test to show the difference in the horizontal ground displacement caused by an inclined and vertical fault for a near field observation. The ground displacement of an inclined fault is asymmetrical and different from that of a vertical fault up to distances approximately 3 times that of the fault’s length (Fig. S1). In contrast to most studies in or around Ordos that assume the faults are vertical^5,13^, we develop an improved method to derive the faults’ geometry parameters under the constraints of geological data and micro-earthquake relocation results. Then, the fault slip rates are inverted by using the dense interseismic GPS velocities with a block model^14–16^ and assuming fully locked faults above the locking depth. After deriving the slip rates of each fault, we move a step further to calculate the rates of moment accumulation that provide a critical input for seismic moment deficit analysis.

## Construction of Fault Block Model

**Figure 1:**
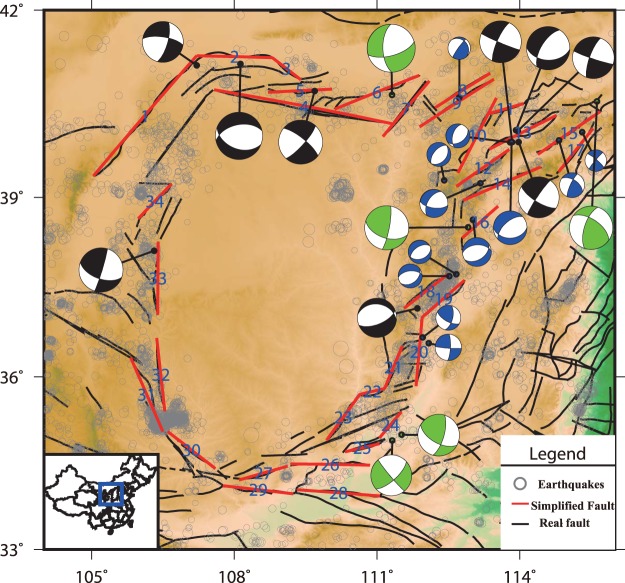
Thirty-four simplified fault segments are represented by red lines. Their dip angles and locking depths are estimated using the earthquake catalogue data. The grey circles represent the micro earthquake events we use. Different colour focal mechanisms represent earthquakes from different earthquake catalogues. The blue symbols are from Cai *et al*. (2013). The green and black symbols are from Gao *et al*.^17^ and gCMT catalogue, respectively.

The earthquake relocation results (Fig. 1) from 2003 to 2012 and an improved method^17,18^ of inverting the dip angle are applied to construct a 3-D fault model of this area (see Methods section). The estimated fault geometry parameters are shown in Fig. 1 and table S1. Many studies have shown that crustal strain is mainly elastic over the short timescale of the GPS observation^19–21^. There are studies^5,13,22–24^ in China suggesting that Ordos is block-like, and some of them also use a block model to compute the block rotation velocity of China. Therefore, we think it is plausible to adopt the block model in Ordos following the previous studies.

**Figure 2:**
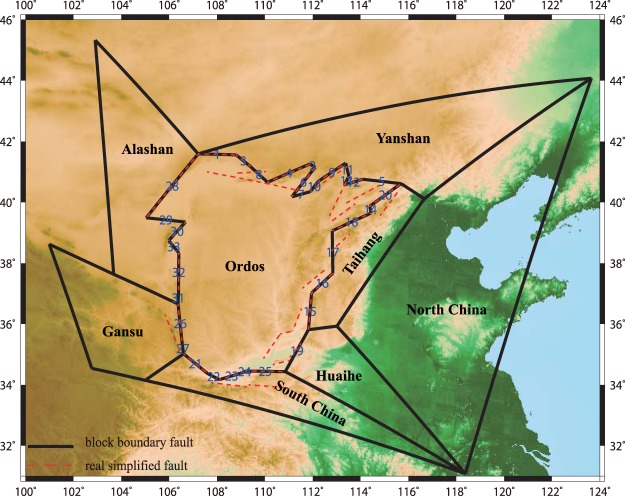
The block model that we constructed is based on the 34 fault segments and previous studies. The dotted red lines are the 34 faults shown in Fig. 1. The surrounding blocks may be smaller than the real blocks because we consider only the part that contains the GPS stations used in our study.

We connect some of the 34 fault traces on the Ordos boundaries and add 7 surrounding blocks that are covered by the GPS data (Fig. 2). We connect the outer faults of Ordos to form the block, which is similar to the study of Wang *et al*.^5^. The sub-parallel fault systems are always represented by one in which the sub-parallel faults in Ordos merge to a single fault in the deep crust^25^. Finally, we obtain 33 faults to enclose the Ordos Block, and we show the block boundaries in Fig. 2 and table S2. We divide the 33 faults into 5 fault systems: Hetao Rift (Faults 1 to 13), Shanxi Basin Rift (Faults 14 to 20), Weihe Rift (Faults 21 to 25), Ningxia Rift (Faults 26 and 27) and Western Ordos (Faults 29 to 33) (see table S2). The faults marked by asterisks in Table S2 are those added to enclose the block. The artificially added faults are assumed to be vertical, and their longitudes and latitudes are shown in table S2. Table S2 also shows the fault relationship between Figs 1 and 2.

## GPS Data

**Figure 3:**
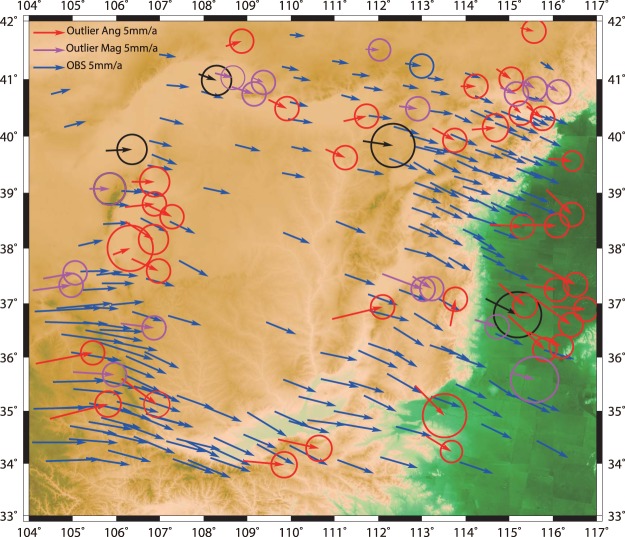
The total 263 GPS velocities are shown with 70% confident error ellipses. The vectors without error ellipses have similar errors to the blue vector with error ellipses whose errors are small. The black vectors are those observations with errors larger than 1.3 mm/yr, either in the east or north direction, but not detected as an outlier. The red and magenta velocities are the outliers whose orientations or magnitudes diverge from nearby GPS velocities. The reference frame is the Eurasia Plate.

The “China Observation Net”, operated by the Second Monitoring Center of the China Earthquake Administration, provides the current consensus estimates of interseismic GPS velocity field from 1999 to 2007 in Ordos, and the sampling rate of GPS stations is 1/30 Hz. We choose the stable Eurasia Plate as the reference frame to express the GPS velocities for a convenient comparison with previous studies. The GPS data processing and outlier picking methods are shown in the Methods section. The 209 GPS station velocities are represented by arrows in Fig. 3. The red and magenta arrows represent velocity outliers.

## Results

We use the fault model, GPS data and the block inversion model to invert for the slip rate of each fault. The predicted slip rates provide profound insights for the seismic hazard analysis. The balance between interseismic elastic strain accumulation and coseismic release defines the extent to which a fault system exhibits a surplus or deficit of strain energy to be released in large earthquakes^26^. In this section, we calculate the fault slip rates and compare the seismic moment accumulation and release in these five fault zones.

### Data fit

**Figure 4:**
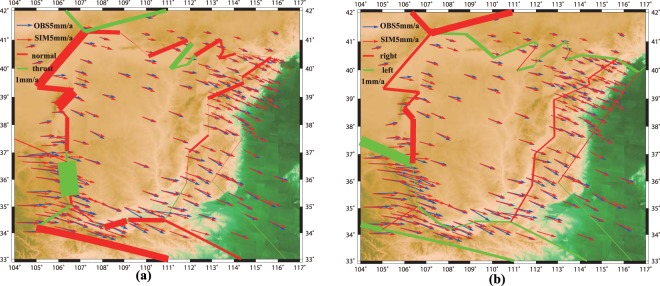
Predicted dip and strike-slip rates are shown in (**a**) and (**b**), respectively. In (**a**), a red line represents a normal fault and a green line represents a thrust fault. In (**b**), a red line and a green line represent right-lateral and left-lateral faults, respectively. The thickness of the lines is proportional to the slip rate. The blue and red arrow represents the observation and predicted GPS data, respectively.

**Figure 5:**
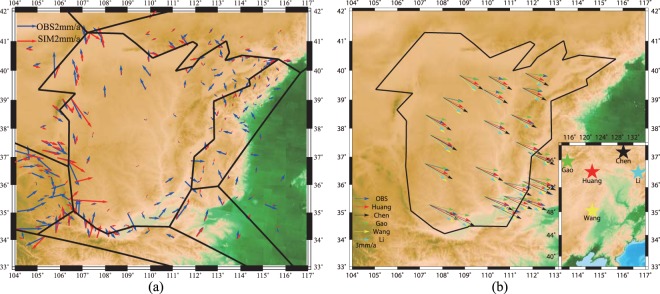
The residual velocity due to faults’ back slip and the block rotation. (**a**) The velocities with the block rotation velocity removed from the observed and predicted GPS velocities (respectively represented by blue and red arrows). (**b**) The locations of Euler pole we derived and the rotation velocity predicted from previous studies.

We use a block modelling code developed by Meade and Loveless^15^ with connected fault-network geometries and a linear elastic earth model, expressed in spherical coordinates, for the inversion. The estimated slip rates are computed from the estimated block motions and are presented in Table S3 and Fig. 4. The predicted velocity field accounts for most features of the observed velocity field. The total sum of squares of the residuals and root-mean-square of the residual are 145.9 (mm/yr)^2^ and 0.7 mm/yr, respectively. The square root of the mean-squared residual is far less than the uncertainty of the GPS data, and 87.56% of the residual velocity components are smaller than their observation uncertainties. The total GPS velocity is modelled as the sum of the block rotation velocity and elastic deformation from the faults. Therefore, if we remove the calculated block rotational velocity from the raw GPS velocity, we can obtain the elastic deformation (shown in Fig. 5a) due to the fault slip deficit. The deformation caused by fault locking only accounts for a minor part of the total velocity field (almost always less than 2 mm/yr). The model fits well for most stations, except some in the south-western corner.

### Improvement from using inclined faults

One strong point of our study is implementing an improved method to estimate the dip angle of faults. We do a blank test to illustrate the effectiveness of our improvement for inversion. In the blank test, we set all the faults vertical and calculate the fault slip rates. The total sum of the square residual is 390 (mm/yr)^2^, which is 2.5 times the result of our method. From this aspect, the estimation of fault dip angle substantially reduces the model misfit.

### Slip Rates on Major Faults

The estimated along-strike and along-dip slip rates are shown in Fig. 4a,b, respectively. We briefly describe the slip rates in these fault zones; the details are listed in Table S3. The results are compared with geological and other geodetic studies. We find that nearly all of the faults share normal-fault motion, except for the 26^*th*^ fault with a significant thrust-fault motion. The strike slip components on the east and west sides are generally dextral, and on the north and south, they are generally sinistral.

In the Hetao Rift, the strike slip is sinistral and ranges from *0.5* to 2 *mm/yr*, except for the 6^*th*^ and 13^*th*^ faults whose slip rates are both −0.5 ± *0.5* *mm/yr* dextral. We also find systematic normal motion ranging from 0.2 to 2.5 mm/yr, except for the 6^*th*^, 7^*th*^ and 10^*th*^ faults.

In the Shanxi Basin Rift, the strike-slip rates are uniformly dextral and small. The normal slip rates generally decrease from north to south. We find that the 19^th^ fault has a 0.5 mm/yr thrust slip rate with an estimated error of 0.8 mm/yr. Given that the estimated error is comparable to its slip rate, it is plausible that this fault has purely normal slip motion.

The faults in the Weihe Rift share common sinistral slip rates of approximately 1 mm/yr. We find a relatively large normal motion in this area, except for the 22^*nd*^ and 24^*th*^ faults. However, it is difficult to confirm whether the two faults represent the real situation because they were added manually to make the Ordos Block enclosed. Therefore, we think the fault motion in this area has sinistral and normal components.

The Ningxia Rift only contains two faults. It is noteworthy that the 26^th^ fault, which marks part of the boundary between the Ordos Block and Tibetan Plateau, has an extremely large thrust motion of approximately *10.6* *mm/yr*, accompanied by a small sinistral slip component. This large thrust motion will be discussed below.

Finally, the faults in the Western Ordos Fault system slip dextrally at rates ranging from *1.5* to *3.4* *mm/yr* with normal slip of *1.2* *mm/yr* to *6.5* *mm/yr*. The mean slip rate in this area is relatively large compared to other fault zones.

The comparison with previous studies is shown in Table S3. Our results mainly agree with other studies in the slip direction^26–29^ but are slightly different in the dip-slip velocities as determined with geological surveys^24^. Fault 1 has a larger dip slip rate than the geological estimate, but Fault 3 has a smaller rate. For the Shanxi Basin Rift (Faults 14 to 20), our results agree with the geological^27,30^ and geodetic^28^ estimates. The magnitude of the dip-slip velocities in the Weihe Rift (Faults 21 to 25) agrees well with other studies; however, the strike-slip rate in our results are smaller than that of geological estimates^31,32^. For the Ningxia (Faults 26 and 27) and Western Ordos (Faults 28 to 33), only a few studies^27,33^ have been done on them; however, the slip direction of our results agrees well with other research. Our result is also supported by the focal mechanisms of 8499 earthquakes in Ordos^34^. The mechanisms of micro-earthquakes in the Southern Fault Zone are mainly left-lateral^35,36^ and this outcome agrees with our results.

### Ordos Euler Pole

The Ordos Block is a relatively rigid block, and many researches^5,17,33,37^ have studied the location of its Euler pole. The results of previous studies and our study are shown in Fig. 5b and Table S4. The locations of the Ordos Euler pole range from 115°N–133°N, 48°E–57°E, and the rotation rate ranges from 0.12 to 0.20 rad/Myr. Our result shows that the Ordos Block rotates counter-clockwise around a Euler pole located at 121.66°*E* ± 4.45° and 54.46°*N* ± 4.31°. The rotation rate is 0.14 ± 0.036*rad*/*Myr*. Our derived result falls in the range of other previous studies. From Fig. 5b, we can see that Gao’s result^17^ fits the data better than the other results. We calculate the total sum of squared residuals and list them in table S4. Our model outperforms previous studies because our result has smaller residuals overall. As to other blocks’ rotation, the uncertainties of the Alashan Block, Yanshan Block North China Plain are relatively larger than their estimated velocities. The location of the Yanshan Block’s Euler pole has the largest uncertainty. The Gansu Block has the largest rotation velocity, which is similar to the results of Wang *et. al*.^5^.

### Moment Balance

**Figure 6:**
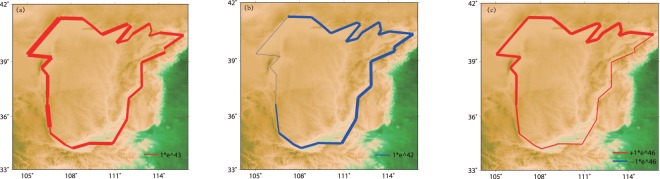
The seismic moment, accumulated and released, are shown in (**a**) and (**b**), respectively; (**c**) shows the moment deficit. The red lines represent the unreleased moment in (**c**). The magnitude of the seismic moment is proportional to the width of the line.

**Figure 7:**
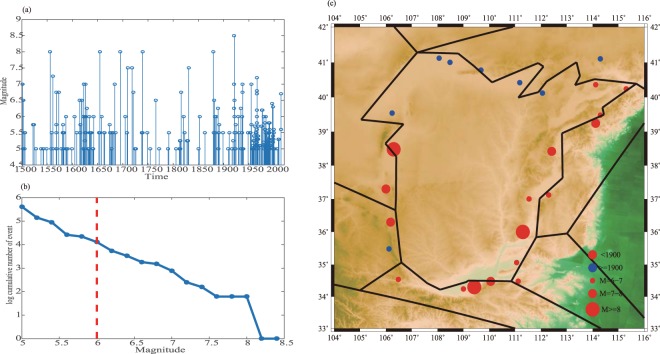
Events with magnitude above 5 versus time are shown in (**a**). The log cumulative earthquake number above a certain magnitude are shown in (**b**). The magnitude of completeness is chosen to be 6.0. The space seismicity from the 1500 s are shown in (**c**). Red and blue dots represent the events that occurred from 1500 to 1900 and after, respectively. The magnitude is proportional to the circle size.

Combining the fault slip rates and the earthquake catalogue, we can calculate the rates of moment accumulation and release of each fault system since the time when the last major earthquake happened (Fig. 6 and table S3). The reference times for the Shanxi Basin Rift, Weihe Rift, Ningxia Rift, and Western Ordos Fault System are taken to be 1696, 1557, 1623, and 1740, respectively based on the time of the last large earthquake in each rift (Fig. S2). All the earthquakes recorded in the Hetao Rift are all under magnitude 7; therefore, we choose the year 1500 as the beginning time after which earthquakes larger than 6 are recorded completely (Fig. 7). The Hetao Rift and Western Ordos Fault system accumulate relatively more seismic moment, while the Shanxi Basin Rift has released the most seismic moment. All of these fault systems have unreleased seismic moments since their latest largest earthquakes. The Hetao Rift has the most unreleased moment, followed by the Western Ordos and Ningxia Rift. We compare the seismic moment deficit with seismicity (Fig. 7c) and find that almost all the earthquakes above M6 after the 1900s struck the Hetao Rift, which is exactly the place with the largest excess seismic moment. One M6 earthquake after 1900 struck the Western Ordos and Ningxia Rift, which also have relatively large unreleased seismic moments compared to those of the Shanxi Basin Rift and Weihe Rift. In contrast, there has been no large earthquakes since 1900 in the other places. Considering these facts, we think the Hetao Rift is the most seismically risky.

## Discussion

Although many studies based on GPS observation have been conducted in China, only a few of them have looked into the Ordos Block. Most studies focus on the Tibetan Plateau^4,12,36^ and southwest China^38,39^. Studies on the whole of China^5,13,35^ include the Ordos Block; however, they are not as specific as this study on the fault geometry and analysis of seismic hazards.

There are limitations and uncertainties of the model and data in this study, as discussed below. The elastic block model assumes that crustal deformation is concentrated along fault zones bounding rigid crustal blocks. There are hardly any faults inside the Ordos Block; thus, the block model is more appropriate for Ordos. We assume the faults to be fully locked above the locking depth; however, this could overestimate the estimated moment accumulation if some fault creep exists. However, the crustal kinematics of a large part of Asia is block or plate-like and continuous internal strain is limited to the Tibetan Plateau^38^.

The viscosity of the Ordos lower crust is above 10^21^ *Pa*·*s*^40^, and the Maxwell relaxation time is estimated to be at least 400 years, while the recurrence interval is less than 400 years from the magnitude-time relationship (Fig. S2). Therefore, ${{\rm{\tau }}}_{0}=\frac{recurrence\,interval}{2\cdot Maxwell\,relaxtion\,time}$ is less than 0.5. The block model and seismic estimation in this study should approximate the real situation. So, it’s reasonable to adapt the elastic half-space block model to approximate the surface deformation and need not to consider the effect of viscoelastic relaxation and a two-layer rheology model^41^ in this study. From another aspect, even the viscous effect in the North Anatolian Fault, which accounts for 23% of the whole crustal deformation, is relatively small compared to our estimated slip rate errors, some of which exceed half the estimated slip rates in our study. Therefore, we think the viscoelastic effect can be neglected considering the large estimated error.

The GPS data contain some outliers (Fig. 3) with large errors and we applied outlier detection (see Methods) to remove them. We find that the clearly wrong observations and observations with large errors are picked out by using our approach of detecting outliers. The root-mean-squared residual reduced from 1.16 mm/yr to 0.84 mm/yr after picking out the 54 outliers. We remind readers that only under the condition that the rotation component is the main component of the GPS velocity can this method be used. According to our calculation (see Fig. 6), the rotation velocity is approximately 80% of the whole velocity. Therefore, we believe that comparing velocities with their neighbours is possible. The criteria we choose for orientation and magnitude will surely vary with different reference frames, and tests should be conducted to determine the criteria of the detection of outliers.

Apart from the counterclockwise rotation around the Euler pole, the properties of slip indicate the dynamics of Ordos. There are many interpretations of the dynamics of Ordos^24,42,43^. According to Deng’s study^24^, under the NE-directed compression from the Tibetan Plateau and the NW-SE extension caused by the centre on the Datong volcanic area, the surrounding faults of Ordos all move with extensional components except for the southwest Ordos and this is in accordance to our results. The strike-slip rates are mainly determined by the motion of the blocks surrounding Ordos. The Tibetan Plateau pushes the Ordos Block from the southwest at the connection of Faults 26 and 27, and the outer walls of Faults 26 and 27 move apart from each other and form dextral and sinistral movements, respectively. Therefore, we can see the faults in the Western Ordos and Weihe Rift have left-lateral and right-lateral motions, respectively. The Ordos rotates counterclockwise around its Euler pole in northeastern China as a whole. Therefore, the Ordos Block moves east and north relative to the Huabei Block, the faults in the Hetao Rift have left-lateral motion, and the faults in the Shanxi Basin Rift have right-lateral motion.

The thrust slip for Fault 26 is extremely large, and we think this condition may be caused by an imperfection in the model assumption. The elastic half space assumption may not hold in the Gansu Block because the Gansu Block is the edge of the Tibetan Plateau, the viscosity in the Tibetan Plateau is relatively low (approximately 10^19^ to 10^20^ Pa·s), and we can see that the deformation is more diffusely distributed (Fig. 5a). The estimation of the fault slip rate will be higher if the deformation is completely attributed to the elastic effects. The assumption of full locking may also not hold in this place.

We have calculated the seismic moment deficit of each fault, but the results should be interpreted with caution. We are fortunate to have 500 years of relatively complete records of M ≥6.0 earthquakes. Meade and Hager^25^ suggest that the catalogue should be long enough to reflect the characteristic seismic activity, and the largest earthquake is included. Earthquakes larger than M 7.0 are not recorded in the Hetao Rift; thus, it is possible that the catalogue for Hetao Rift is not long enough. However, considering the evidence that there are continuous *M*_*w*_ 6 to 7 earthquakes occurring in the north from 1900, it indicates that these faults may be in an active stage to which attention should be paid. The moment deficit is a lumped sum for all faults in a fault zone, whereas earthquakes occur on an individual fault. We cannot predict the exact place where an earthquake will happen. The result of the moment deficit in Ordos can only provide some guidance for future studies and alert people where there is a high earthquake risk.

## Methods

### Estimation of fault parameters

When estimating the dip angle of a fault, we first select the earthquake events whose epicentres are less than 50 *km* from the fault surface trace. We assume the dip angle of the fault plane is *δ*, and let *d*_*i*_ denote the perpendicular distance of the *i*_*th*_ earthquake to the fault plane. Then, we change the dip angle *δ* from 0° to 90° and choose the one that maximizes the following likelihood function as the fault’s dip angle:

1 $$P(\delta )=\sum _{i=1}^{n}log\{[\frac{{\sum }_{i=1}^{n}\frac{1}{{\sigma }_{i}}}{{\sigma }_{i}\sqrt{2\pi }}{\exp }(-\frac{{d}_{i}^{2}}{2{{\sigma }_{i}}^{2}})]{\omega }_{i}+\alpha \};$$

where σ_*i*_ is the estimated error of the depths of earthquakes (we assume the location error of each earthquake is normally distributed); *ω*_*i*_ is a weight factor proportional to the square of earthquake magnitude; and α is a constant (usually *0.1*) that prevents the first term in the parentheses from being zero. We show the inversion result for the Taigu Fault in Fig. S3 as an example.

The depth that separates the 95% shallowest and 5% deepest earthquakes is taken as the locking depth of a fault in many studies. Because the location of small earthquakes has a large uncertainty, and many earthquakes are hard to be classified as being on a single fault, we estimate a locking depth for a whole fault system instead (Fig. S4).

### GPS data processing and outlier picking

The GAMIT software^44^ was used to obtain loosely constrained daily solutions for satellite orbits and positions of regional stations. Approximately ninety evenly distributed global ITRF GPS core stations were also processed using GAMIT. Then, using GLOBK software^45^, the daily solution for regional stations was combined with the loosely constrained solutions of the ITRF core stations. The QOCA software^46^ was employed to estimate the station positions and velocities within the ITRF2008 reference frame^47^. Finally, the velocities were transformed into a Eurasia-fixed reference frame. We only use the east and north components due to the large uncertainties of the vertical component.

There are 263 stations in total; however, some of the outliers—due to various nontectonic sources—should be excluded (data are shown in table S5). We assume the rotation velocity is the major component of the GPS velocity, and the velocities’ vector orientations and magnitudes should change slightly in a certain area on the same block. Therefore, we can compare each GPS observation’s vector orientation and magnitude with other GPS observations within a radius of 0.75° (the number of data is sufficient to detect outliers in this area) to detect outliers. The azimuth differences and magnitude ratios of each GPS velocity to its nearby velocities are calculated. If the median of the azimuth differences is larger than 12°, or the median of the velocity ratios is smaller than 0.75 or larger than 1.25, which means the GPS velocity is clearly different from its neighbouring velocities in orientation or magnitude, this GPS velocity will be taken as an outlier. We have two main standards for choosing the threshold to detect outliers. The first standard is that the number of left GPS data used for the inversion should be moderate. A tight threshold leads to more outliers detected and vice versa. The second standard is that the inversion results should not be sensitive to the data selected by the threshold. We test other thresholds around the one we choose (12° for azimuths and 0.75/1.25 for magnitudes) and find our thresholds are stable. The outliers are shown in Fig. 3 in detail. After selecting the GPS data, 209 GPS velocities are left and are used in the inversion of the fault slip rates. We remind readers that our approach of detecting outliers by comparing each observation to its nearby observations cannot give convincing results when the data are sparse in space. However, as demonstrated in Fig. 3, the data are sparse only in the northwest corner and inside the Ordos Block.

### Moment calculation

The rate of scalar moment (*M*_0_) accumulation on a given fault is estimated as follows:

2 $${\dot{M}}_{0}=\mu \dot{D}A;$$

where *μ* is the shear modulus that we assume as 30 *GPa; D* is the total slip rate, including both the strike-slip and dip-slip rates; and *A* is the fault area, taken as the product of the fault width and the fault length. There is a simple and straightforward empirical law^48^ between the moment magnitude (*M*_*w*_) and the seismic moment (*M*_0_):

3 $${M}_{w}=\frac{2}{3}\,{log}\,{M}_{0}-6.03;$$

where M_0_ is in N m. We choose *M*_*C*_ = 6.0 as the magnitude of completeness, for the number of events larger than M_w_ 5.0 is higher in recent years (Fig. 7a), likely due to the improvement of seismic station coverage. The slope between the magnitude and earthquake frequency become stable above magnitude 6 (Fig. 7b). The studies^49,50^ both showed that the catalogue for M ≥ 6 is likely complete since 1300 in North China. Meade and Hager^15^ compared the seismic moment release due to small and large earthquakes. We follow their method and find that all the earthquakes up to *M*_*w*_ = 6.0 only account for 10% of the total moment release (in the calculation, we set the *b* value in the G-R law to be 1). Other studies^51^ showed a similar conclusion. Hence, we select a relatively high magnitude of completeness when dealing with the historical earthquake catalogue from the 1500 s. The moment release from events below *M*_*C*_ is not accounted for quantitatively but is expected to be small. We subtract the released seismic moment from the accumulated seismic moment to obtain the seismic deficit for each fault system.

## Electronic supplementary material

Supplementary materials
