# Supplementary material for: Fault Slip Rates and Seismic Moment Deficits on Major Faults in Ordos Constrained by GPS Observation
Source: Sci Rep. 2018 Nov 1;8:16192. doi: 10.1038/s41598-018-34586-2 (PMC6212509; doi:10.1038/s41598-018-34586-2)
Supplement: Supplementary file 1 — Supplementary materials [file 41598_2018_34586_MOESM1_ESM.docx]

Fault Slip Rates and Seismic Moment Deficits on Major Faults in Ordos Constrained by GPS Observation

Yilei Huang^1)^, Qingliang Wang^2)^, Ming Hao^2)^ and Shiyong Zhou^1)^

1. Institute of Theoretical and Applied Geophysics, Peking University, Beijing, 100871, China
2. Second Monitoring Center, China Earthquake Administration, Xi'an, 710043, China

*Corresponding to Shiyong Zhou, email: [zsy@pku.edu.cn](mailto:zsy@pku.edu.cn)

**Supplementary Materials**

Table S1 Parameters of faults around Ordos

Table S2 Relationship between faults in Fig. 2 and Fig. 3

|  | Identifier in Fig 3 | Identifier in Fig 2 | Lat1 | Lon1 | Lat2 | Lon2 |
| --- | --- | --- | --- | --- | --- | --- |
| Hetao Rift | 1 | B |  |  |  |  |
|  | 2* |  | 111.89 | 40.98 | 112.10 | 40.88 |
|  | 3 | C |  |  |  |  |
|  | 4 | F |  |  |  |  |
|  | 5* |  | 114.08 | 40.52 | 115.69 | 40.39 |
|  | 6 | G |  |  |  |  |
|  | 7* |  | 111.12 | 39.99 | 111.90 | 40.15 |
|  | 8* |  | 109.40 | 40.90 | 110.11 | 40.43 |
|  | 9 | H |  |  |  |  |
|  | 10* |  | 111.90 | 40.15 | 112.22 | 40.45 |
|  | 11* |  | 113.38 | 41.01 | 113.51 | 40.61 |
|  | 12 | K |  |  |  |  |
|  | 13* |  | 113.32 | 40.35 | 113.51 | 40.61 |
| Shanxi Basin Rift | 14* |  | 114.38 | 39.70 | 114.44 | 39.50 |
|  | 15 | T |  |  |  |  |
|  | 16 | S |  |  |  |  |
|  | 17* |  | 112.82 | 38.95 | 112.84 | 37.63 |
|  | 18 | N |  |  |  |  |
|  | 19* |  | 110.85 | 34.48 | 111.82 | 35.84 |
|  | 20 | O |  |  |  |  |
| Weihe Rift | 21 | AD |  |  |  |  |
|  | 22* |  | 107.60 | 34.42 | 108.10 | 34.22 |
|  | 23 | AA |  |  |  |  |
|  | 24* |  | 109.12 | 34.46 | 109.18 | 34.50 |
|  | 25 | Z |  |  |  |  |
| Ningxia Rift | 26 | AF |  |  |  |  |
|  | 27* |  | 106.54 | 35.41 | 106.60 | 35.06 |
| Western Ordos | 28 | A |  |  |  |  |
|  | 29* |  | 105.04 | 39.35 | 106.68 | 39.22 |
|  | 30 | AG |  |  |  |  |
|  | 31* |  | 106.37 | 36.66 | 106.39 | 37.05 |
|  | 32 | AH |  |  |  |  |
|  | 33* |  | 105.98 | 38.65 | 106.40 | 38.27 |

* the fault with * is the added fault to construct the closed Ordos block.

Table S3. Slip rates and Moment Balance on Ordos

^a^ Positive for sinistral motion and negative for dextral motion

^b^ Geodetic references: (1)^36^; (4)^30^; (5)^31^; (8)^35^. Geological references:(2)^32^;(3) ^29^; (6)^33^; (7)^34;^

^c^ Positive for normal motion (extension) and negative for thrust motion (contraction). If the fault is vertical, extension or contraction represent the horizontal motion away from or toward the fault.

^*^ Faults are added manually to form a closed fault system and the dip angles are 90$^{\circ}$.

Table. S4 Euler poles of Ordos and surrounding block.

|  | Model | Euler Lon (°) | | Euler Lat (°) | | Rotation Rate (rad/Myr) | | Total sum of residual (mm/yr)^2^ |
| --- | --- | --- | --- | --- | --- | --- | --- | --- |
| Ordos Block | Huang et al. (this paper) | 121.66 | (4.45) | 54.46 | (4.31) | 0.14 | (0.04) | 8.4 |
|  | Wang et al., 2011 | 121.67 |  | 48.17 |  | 0.20 |  | 9.47 |
|  | Li et al, 2003 | 133.20 |  | 54.20 |  | 0.12 |  | 11.3 |
|  | Gao et al., 2016 | 115.48 |  | 55.82 |  | 0.13 |  | 12.98 |
|  | Chen et al., 2011 | 129.50 |  | 57.10 |  | 0.14 |  | 36.15 |
| Alashan Block |  | 101.99 | (4.84) | 48.14 | (10.86) | 0.17 | (0.18) |  |
| Yanshan Block |  | 282.67 | (22.51) | -15.97 | (63.18) | 0.05 | (0.11) |  |
| Taihang Block |  | 125.25 | (7.42) | 51.17 | (6.52) | 0.17 | (0.09) |  |
| South China Block |  | 123.54 | (15.23) | 53.20 | (11.64) | 0.18 | (0.11) |  |
| Gansu Block |  | 284.05 | (0.48) | -29.66 | (1.71) | 0.64 | (0.17) |  |
| North China Plain |  | 127.20 | (17.90) | 50.41 | (15.16) | 0.16 | (0.17) |  |
| Huaihe Block |  | 217.36 | (23.97) | 64.70 | (8.54) | 0.06 | (0.06) |  |

^*^ the data in parentheses are the uncertainty of calculation

^*^the reference frame for all the Euler poles is the stable Eurasia Plate.

Table S5. The observed and simulated GPS velocity

| Station Name | Lon° | Lat° | N_comp (mm/yr) | | E_comp (mm/yr) | | Estimated N_comp (mm/yr) | Estimated E_comp (mm/yr) | N_residual (mm/yr) | E_residual （mm/yr） |
| --- | --- | --- | --- | --- | --- | --- | --- | --- | --- | --- |
| A001 | 112.61 | 41.27 | -0.4 | (1.2) | 2.6 | (1.2) | -0.6 | 2.5 | 0.2 | 0.1 |
| A002 | 112.56 | 40.89 | -0.5 | (1.2) | 2.5 | (1.2) | -0.9 | 2.7 | 0.4 | -0.2 |
| A004 | 112.35 | 40.17 | -1.2 | (1.2) | 4.1 | (1.2) | -1.1 | 3.7 | -0.1 | 0.4 |
| A005 | 113.13 | 41.02 | -0.3 | (1.2) | 2.6 | (1.2) | -1.0 | 2.6 | 0.7 | 0.0 |
| A006 | 113.21 | 40.79 | -0.5 | (1.2) | 2.9 | (1.2) | -1.3 | 3.1 | 0.8 | -0.2 |
| A007 | 113.20 | 40.45 | -0.5 | (1.2) | 3.6 | (1.2) | -1.1 | 3.4 | 0.6 | 0.2 |
| A008 | 112.73 | 40.02 | -1.3 | (1.2) | 4.5 | (1.2) | -1.2 | 3.9 | -0.1 | 0.6 |
| A010 | 113.99 | 41.11 | -0.8 | (1.2) | 2.5 | (1.2) | -0.8 | 2.5 | 0.0 | 0.0 |
| A012 | 113.76 | 40.37 | -1.3 | (1.2) | 2.8 | (1.2) | -1.5 | 3.5 | 0.2 | -0.7 |
| A013 | 113.66 | 40.04 | -1.8 | (1.2) | 4.2 | (1.2) | -1.2 | 3.9 | -0.6 | 0.3 |
| A015 | 114.22 | 40.71 | -1.2 | (1.2) | 2.9 | (1.2) | -0.9 | 2.7 | -0.3 | 0.2 |
| A016 | 114.43 | 40.45 | -1.2 | (1.2) | 3.2 | (1.2) | -1.1 | 3.3 | -0.1 | -0.1 |
| A017 | 114.12 | 40.46 | -1.7 | (1.2) | 3.7 | (1.2) | -1.1 | 3.4 | -0.6 | 0.3 |
| A021 | 114.82 | 40.44 | -1.5 | (1.2) | 3.8 | (1.2) | -1.0 | 3.2 | -0.5 | 0.6 |
| A022 | 114.68 | 40.23 | -1.4 | (1.2) | 3.6 | (1.2) | -1.1 | 3.7 | -0.3 | -0.1 |
| A023 | 114.95 | 40.09 | -1.6 | (1.2) | 3.8 | (1.2) | -1.4 | 3.9 | -0.2 | -0.1 |
| A024 | 112.84 | 39.82 | -1.2 | (1.2) | 4.8 | (1.2) | -1.2 | 4.0 | 0.0 | 0.8 |
| A025 | 112.81 | 39.54 | -2.1 | (1.2) | 3.5 | (1.2) | -1.3 | 4.1 | -0.8 | -0.6 |
| A026 | 112.84 | 39.18 | -1.6 | (1.2) | 4.5 | (1.2) | -1.4 | 4.3 | -0.2 | 0.2 |
| A027 | 112.80 | 38.99 | -1.6 | (1.2) | 4.4 | (1.2) | -1.6 | 4.6 | 0.0 | -0.2 |
| A028 | 112.77 | 38.73 | -1.6 | (1.2) | 4.6 | (1.2) | -1.6 | 4.3 | 0.0 | 0.3 |
| A029 | 112.69 | 38.40 | -1.8 | (1.2) | 4.7 | (1.2) | -1.6 | 4.4 | -0.2 | 0.3 |
| A030 | 113.06 | 39.82 | -0.9 | (1.2) | 4.4 | (1.2) | -1.2 | 4.0 | 0.3 | 0.4 |
| A031 | 113.28 | 39.58 | -2.1 | (1.2) | 4.3 | (1.2) | -1.3 | 4.1 | -0.8 | 0.2 |
| A032 | 113.19 | 39.18 | -2.4 | (1.2) | 4.6 | (1.2) | -1.7 | 4.4 | -0.7 | 0.2 |
| A033 | 113.66 | 39.06 | -2 | (1.2) | 4.3 | (1.2) | -2.0 | 4.2 | 0.0 | 0.1 |
| A035 | 112.97 | 38.44 | -1.9 | (1.2) | 3.8 | (1.2) | -1.9 | 4.3 | 0.0 | -0.5 |
| A036 | 113.64 | 39.72 | -1.6 | (1.2) | 4.5 | (1.2) | -1.2 | 4.1 | -0.4 | 0.4 |
| A037 | 113.81 | 39.57 | -1.8 | (1.2) | 3.9 | (1.2) | -1.3 | 4.1 | -0.5 | -0.2 |
| A038 | 113.74 | 39.29 | -1.3 | (1.2) | 4.5 | (1.2) | -1.9 | 4.3 | 0.6 | 0.2 |
| A039 | 113.94 | 39.11 | -1.3 | (1.2) | 4.4 | (1.2) | -2.0 | 4.1 | 0.7 | 0.3 |
| A040 | 113.64 | 38.78 | -2.2 | (1.2) | 4.2 | (1.2) | -2.0 | 4.2 | -0.2 | 0.0 |
| A041 | 114.22 | 39.77 | -1.4 | (1.2) | 4.4 | (1.2) | -1.3 | 4.1 | -0.1 | 0.3 |
| A042 | 114.23 | 39.43 | -1.6 | (1.2) | 4.2 | (1.2) | -2.0 | 4.2 | 0.4 | 0.0 |
| A043 | 114.23 | 38.88 | -2 | (1.2) | 3.9 | (1.2) | -1.9 | 4.1 | -0.1 | -0.2 |
| A044 | 114.12 | 38.58 | -1.6 | (1.2) | 4.7 | (1.2) | -2.0 | 4.2 | 0.4 | 0.5 |
| A045 | 115.27 | 39.64 | -1.5 | (1.2) | 3.6 | (1.2) | -1.7 | 3.8 | 0.2 | -0.2 |
| A046 | 114.58 | 39.84 | -1.6 | (1.2) | 4.2 | (1.2) | -1.6 | 4.2 | 0.0 | 0.0 |
| A047 | 114.82 | 39.70 | -1.6 | (1.2) | 3.9 | (1.2) | -1.8 | 3.9 | 0.2 | 0.0 |
| A048 | 114.56 | 39.36 | -1.5 | (1.2) | 4.2 | (1.2) | -1.8 | 4.1 | 0.3 | 0.1 |
| A049 | 114.74 | 39.04 | -1.7 | (1.2) | 4.5 | (1.2) | -1.8 | 4.0 | 0.1 | 0.5 |
| A050 | 114.58 | 38.83 | -2.1 | (1.2) | 4.4 | (1.2) | -1.9 | 4.1 | -0.2 | 0.3 |
| A052 | 114.93 | 38.77 | -1.9 | (1.2) | 4.1 | (1.2) | -1.8 | 4.1 | -0.1 | 0.0 |
| A054 | 115.41 | 40.69 | -0.7 | (1.2) | 2.7 | (1.2) | -1.0 | 2.6 | 0.3 | 0.1 |
| A056 | 115.17 | 40.34 | -1.5 | (1.2) | 3.9 | (1.2) | -1.1 | 3.4 | -0.4 | 0.5 |
| A057 | 115.16 | 40.19 | -1.3 | (1.2) | 3.5 | (1.2) | -1.4 | 3.8 | 0.1 | -0.3 |
| A058 | 115.72 | 41.30 | -1.3 | (1.2) | 2.1 | (1.2) | -0.9 | 2.5 | -0.4 | -0.4 |
| A061 | 115.60 | 40.39 | -1.1 | (1.2) | 2.9 | (1.2) | -1.3 | 3.1 | 0.2 | -0.2 |
| A062 | 115.48 | 40.35 | -1.5 | (1.2) | 3 | (1.2) | -1.3 | 3.4 | -0.2 | -0.4 |
| A066 | 115.93 | 40.35 | -1.3 | (1.2) | 2.9 | (1.2) | -1.1 | 2.9 | -0.2 | 0.0 |
| A082 | 115.70 | 40.00 | -1.6 | (1.2) | 3.4 | (1.2) | -1.6 | 3.6 | 0.0 | -0.2 |
| A083 | 115.56 | 39.64 | -1.5 | (1.2) | 4 | (1.2) | -1.7 | 3.8 | 0.2 | 0.2 |
| A084 | 115.17 | 39.44 | -1.7 | (1.2) | 4.2 | (1.2) | -1.7 | 3.9 | 0.0 | 0.3 |
| A085 | 115.49 | 39.37 | -1.9 | (1.2) | 4.6 | (1.2) | -1.7 | 3.9 | -0.2 | 0.7 |
| A086 | 115.27 | 39.03 | -1.8 | (1.2) | 4.5 | (1.2) | -1.8 | 4.0 | 0.0 | 0.5 |
| A087 | 115.49 | 38.81 | -1.8 | (1.2) | 4.3 | (1.2) | -1.8 | 3.9 | 0.0 | 0.4 |
| C006 | 114.65 | 38.33 | -1.4 | (1.2) | 3.8 | (1.2) | -1.9 | 4.2 | 0.5 | -0.4 |
| C012 | 114.90 | 37.62 | -1.7 | (1.2) | 3.9 | (1.2) | -2.1 | 4.2 | 0.4 | -0.3 |
| C013 | 114.40 | 37.87 | -1.8 | (1.2) | 3.6 | (1.2) | -2.0 | 4.3 | 0.2 | -0.7 |
| C014 | 113.94 | 38.34 | -1.7 | (1.2) | 3.6 | (1.2) | -2.0 | 4.3 | 0.3 | -0.7 |
| C015 | 114.18 | 38.22 | -2.3 | (1.2) | 3.7 | (1.2) | -2.0 | 4.3 | -0.3 | -0.6 |
| C016 | 114.36 | 38.03 | -1.8 | (1.2) | 3.8 | (1.2) | -2.0 | 4.3 | 0.2 | -0.5 |
| C017 | 113.61 | 37.83 | -2.1 | (1.2) | 3.5 | (1.2) | -2.1 | 4.4 | 0.0 | -0.9 |
| C024 | 114.46 | 37.08 | -2.5 | (2.3) | 5 | (2.4) | -2.2 | 4.3 | -0.3 | 0.7 |
| C025 | 113.37 | 37.09 | -2 | (1.2) | 3.6 | (1.2) | -2.1 | 4.6 | 0.1 | -1.0 |
| C030 | 114.68 | 36.44 | -1.8 | (1.2) | 4.4 | (1.2) | -2.2 | 4.5 | 0.4 | -0.1 |
| C036 | 115.79 | 36.14 | -1.5 | (1.2) | 4.8 | (1.2) | -2.0 | 4.5 | 0.5 | 0.3 |
| C038 | 114.54 | 35.57 | -2.7 | (1.2) | 4.1 | (1.2) | -2.2 | 4.7 | -0.5 | -0.6 |
| C040 | 113.78 | 35.50 | -2.3 | (1.2) | 4.9 | (1.2) | -2.4 | 4.8 | 0.1 | 0.1 |
| C041 | 113.79 | 36.01 | -1.7 | (1.2) | 4.7 | (1.2) | -2.3 | 4.6 | 0.6 | 0.1 |
| C042 | 113.44 | 36.21 | -2.2 | (1.2) | 4.5 | (1.2) | -2.3 | 4.7 | 0.1 | -0.2 |
| C044 | 115.42 | 35.25 | -2.2 | (1.2) | 5.1 | (1.2) | -2.0 | 4.8 | -0.2 | 0.3 |
| C045 | 115.09 | 35.29 | -2.8 | (1.2) | 5.9 | (1.2) | -2.1 | 4.8 | -0.7 | 1.1 |
| C046 | 114.17 | 35.17 | -2.5 | (1.2) | 4.6 | (1.2) | -2.3 | 4.9 | -0.2 | -0.3 |
| C047 | 113.21 | 35.27 | -1.7 | (1.2) | 4.7 | (1.2) | -2.2 | 5.3 | 0.5 | -0.6 |
| C049 | 115.63 | 34.44 | -2.7 | (1.3) | 5 | (1.3) | -1.9 | 5.1 | -0.8 | -0.1 |
| C050 | 114.36 | 34.75 | -2.3 | (1.2) | 4.8 | (1.2) | -2.1 | 5.3 | -0.2 | -0.5 |
| C052 | 112.41 | 34.70 | -1.5 | (1.2) | 5.6 | (1.2) | -2.2 | 5.5 | 0.7 | 0.1 |
| C053 | 113.86 | 34.03 | -1.8 | (1.3) | 5.3 | (1.3) | -2.1 | 5.6 | 0.3 | -0.3 |
| C055 | 112.05 | 34.12 | -1.7 | (1.3) | 5.9 | (1.3) | -1.7 | 6.0 | 0.0 | -0.1 |
| D001 | 110.95 | 41.31 | -0.4 | (1.2) | 2.6 | (1.2) | -0.4 | 2.6 | 0.0 | 0.0 |
| D002 | 111.41 | 41.09 | -0.3 | (1.2) | 2.8 | (1.2) | -0.7 | 2.6 | 0.4 | 0.2 |
| D003 | 110.06 | 41.03 | -0.5 | (1.2) | 2.7 | (1.2) | -0.4 | 2.7 | -0.1 | 0.0 |
| D004 | 111.68 | 40.86 | -0.5 | (1.2) | 3 | (1.2) | -1.0 | 2.8 | 0.5 | 0.2 |
| D005 | 111.16 | 40.72 | -0.8 | (1.2) | 3 | (1.2) | -1.2 | 3.0 | 0.4 | 0.0 |
| D007 | 109.98 | 40.60 | -0.7 | (1.2) | 2.9 | (1.2) | -0.9 | 3.2 | 0.2 | -0.3 |
| D008 | 111.78 | 40.36 | -0.5 | (1.2) | 3.7 | (1.2) | -0.6 | 2.9 | 0.1 | 0.8 |
| D010 | 111.65 | 39.92 | -0.7 | (2.2) | 4.7 | (2.2) | -1.0 | 3.8 | 0.3 | 0.9 |
| D012 | 112.26 | 39.61 | -1.8 | (1.2) | 4.5 | (1.2) | -1.2 | 4.1 | -0.6 | 0.4 |
| D013 | 111.52 | 39.42 | -0.8 | (1.2) | 4.2 | (1.2) | -1.3 | 4.2 | 0.5 | 0.0 |
| D014 | 111.23 | 37.53 | -1.7 | (1.2) | 4.8 | (1.2) | -1.5 | 4.7 | -0.2 | 0.1 |
| D015 | 113.33 | 38.29 | -2.1 | (1.2) | 4.2 | (1.2) | -2.0 | 4.3 | -0.1 | -0.1 |
| D016 | 113.15 | 37.91 | -2.4 | (1.2) | 4.1 | (1.2) | -2.1 | 4.5 | -0.3 | -0.4 |
| D017 | 112.60 | 38.00 | -1.3 | (1.2) | 4.9 | (1.2) | -1.5 | 4.5 | 0.2 | 0.4 |
| D019 | 112.48 | 37.25 | -1.7 | (1.2) | 4.5 | (1.2) | -2.0 | 4.9 | 0.3 | -0.4 |
| D022 | 111.77 | 37.16 | -1.7 | (1.2) | 4.7 | (1.2) | -1.5 | 4.9 | -0.2 | -0.2 |
| D023 | 112.82 | 36.82 | -2.8 | (1.2) | 4.6 | (1.2) | -2.1 | 4.8 | -0.7 | -0.2 |
| D024 | 111.81 | 36.86 | -1.6 | (1.2) | 6.2 | (1.2) | -1.6 | 5.0 | 0.0 | 1.2 |
| D025 | 112.35 | 36.56 | -3 | (1.2) | 4.8 | (1.2) | -2.1 | 4.9 | -0.9 | -0.1 |
| D026 | 112.23 | 36.15 | -2.8 | (1.2) | 4.6 | (1.2) | -2.2 | 5.1 | -0.6 | -0.5 |
| D027 | 111.77 | 36.26 | -2 | (1.2) | 5.7 | (1.2) | -1.7 | 5.1 | -0.3 | 0.6 |
| D029 | 111.54 | 36.13 | -2.8 | (1.2) | 6.2 | (1.2) | -1.6 | 5.1 | -1.2 | 1.1 |
| D030 | 110.68 | 36.09 | -1.2 | (1.2) | 5.8 | (1.2) | -1.6 | 5.1 | 0.4 | 0.7 |
| D031 | 112.20 | 35.70 | -3.1 | (1.2) | 5.4 | (1.2) | -2.2 | 5.2 | -0.9 | 0.2 |
| D032 | 111.33 | 35.55 | -1.7 | (1.2) | 5.6 | (1.2) | -1.6 | 5.3 | -0.1 | 0.3 |
| D033 | 110.69 | 35.60 | -1.3 | (1.2) | 6 | (1.2) | -1.6 | 5.3 | 0.3 | 0.7 |
| D034 | 111.67 | 35.29 | -2.5 | (1.2) | 5.1 | (1.2) | -2.1 | 5.3 | -0.4 | -0.2 |
| D035 | 110.98 | 35.00 | -1.2 | (1.3) | 6.2 | (1.3) | -1.7 | 5.5 | 0.5 | 0.7 |
| D036 | 110.18 | 35.27 | -1.5 | (1.2) | 5.9 | (1.2) | -1.6 | 5.4 | 0.1 | 0.5 |
| D037 | 111.15 | 34.75 | -1.1 | (1.3) | 6.4 | (1.3) | -1.8 | 5.4 | 0.7 | 1.0 |
| D038 | 110.38 | 34.83 | -0.8 | (1.3) | 6.1 | (1.3) | -1.7 | 5.5 | 0.9 | 0.6 |
| D039 | 111.07 | 34.07 | -2.1 | (1.3) | 5.7 | (1.3) | -2.3 | 6.3 | 0.2 | -0.6 |
| D040 | 109.97 | 41.77 | -0.2 | (1.2) | 2.4 | (1.2) | 0.8 | 3.6 | -1.0 | -1.2 |
| D042 | 106.98 | 41.43 | -0.4 | (1.2) | 2.7 | (1.2) | -0.1 | 4.1 | -0.3 | -1.4 |
| D043 | 109.80 | 41.22 | -0.6 | (1.2) | 2.7 | (1.2) | -0.3 | 2.7 | -0.3 | 0.0 |
| D046 | 107.89 | 41.09 | -1 | (1.5) | 2.8 | (1.5) | -1.0 | 3.3 | 0.0 | -0.5 |
| D047 | 107.15 | 40.90 | -0.3 | (1.2) | 3.6 | (1.2) | -1.2 | 4.2 | 0.9 | -0.6 |
| D050 | 107.95 | 40.79 | -1.1 | (1.2) | 3.5 | (1.2) | -1.2 | 3.7 | 0.1 | -0.2 |
| D052 | 109.94 | 40.30 | -0.6 | (1.2) | 3.9 | (1.2) | -1.3 | 3.8 | 0.7 | 0.1 |
| D053 | 107.00 | 40.32 | -1 | (1.2) | 3.8 | (1.2) | -1.4 | 3.9 | 0.4 | -0.1 |
| D054 | 110.01 | 39.82 | -0.7 | (1.2) | 3.2 | (1.2) | -1.5 | 4.0 | 0.8 | -0.8 |
| D055 | 108.72 | 39.85 | -1 | (1.2) | 4 | (1.2) | -1.6 | 4.1 | 0.6 | -0.1 |
| D056 | 109.80 | 39.35 | -0.7 | (1.2) | 3.6 | (1.2) | -1.6 | 4.2 | 0.9 | -0.6 |
| D057 | 108.00 | 39.09 | -0.9 | (1.2) | 4.2 | (1.2) | -1.8 | 4.3 | 0.9 | -0.1 |
| D058 | 109.82 | 38.29 | -1.7 | (1.2) | 4.2 | (1.2) | -1.6 | 4.5 | -0.1 | -0.3 |
| D059 | 107.23 | 38.49 | -1.9 | (1.2) | 4 | (1.2) | -1.9 | 4.6 | 0.0 | -0.6 |
| D061 | 109.43 | 36.65 | -1.9 | (1.2) | 4.7 | (1.2) | -1.7 | 5.0 | -0.2 | -0.3 |
| D062 | 107.19 | 36.77 | -1.5 | (1.3) | 5.2 | (1.3) | -1.9 | 5.1 | 0.4 | 0.1 |
| D064 | 109.82 | 35.59 | -2 | (1.2) | 5.2 | (1.2) | -1.7 | 5.3 | -0.3 | -0.1 |
| D065 | 107.39 | 35.29 | -2.1 | (1.3) | 5.8 | (1.3) | -2.0 | 5.6 | -0.1 | 0.2 |
| D066 | 109.00 | 34.97 | -3.8 | (1.3) | 6.2 | (1.3) | -1.8 | 5.6 | -2.0 | 0.6 |
| D068 | 109.63 | 34.99 | -2.5 | (1.3) | 5.6 | (1.3) | -1.8 | 5.5 | -0.7 | 0.1 |
| D069 | 109.95 | 34.81 | -1.9 | (1.3) | 6.4 | (1.3) | -1.8 | 5.6 | -0.1 | 0.8 |
| D071 | 108.91 | 34.55 | -2.2 | (1.3) | 5.2 | (1.3) | -2.3 | 5.9 | 0.1 | -0.7 |
| D072 | 108.23 | 34.50 | -2.9 | (1.3) | 6.4 | (1.3) | -2.0 | 6.0 | -0.9 | 0.4 |
| D073 | 107.58 | 34.43 | -2.6 | (1.3) | 5.4 | (1.3) | -2.2 | 6.2 | -0.4 | -0.8 |
| D074 | 107.38 | 34.47 | -1.7 | (1.3) | 6.9 | (1.3) | -2.3 | 6.3 | 0.6 | 0.6 |
| D075 | 109.97 | 34.35 | -3.1 | (1.3) | 6 | (1.3) | -2.4 | 6.3 | -0.7 | -0.3 |
| D077 | 108.19 | 34.30 | -2.8 | (1.3) | 6.1 | (1.3) | -2.5 | 6.5 | -0.3 | -0.4 |
| D080 | 108.16 | 34.11 | -2.2 | (1.3) | 6.3 | (1.3) | -2.4 | 6.7 | 0.2 | -0.4 |
| D081 | 107.64 | 34.07 | -2.8 | (1.3) | 6.7 | (1.3) | -1.9 | 7.0 | -0.9 | -0.3 |
| D082 | 107.29 | 34.09 | -2.3 | (1.3) | 6.5 | (1.3) | -1.9 | 7.1 | -0.4 | -0.6 |
| D083 | 106.75 | 39.92 | -0.9 | (1.2) | 3.6 | (1.2) | -1.5 | 4.1 | 0.6 | -0.5 |
| D084 | 106.81 | 39.68 | -1.6 | (1.2) | 4 | (1.2) | -1.6 | 4.2 | 0.0 | -0.2 |
| D085 | 105.76 | 39.75 | 0.2 | (1.5) | 4.1 | (1.5) | -1.0 | 4.7 | 1.2 | -0.6 |
| D086 | 106.72 | 39.49 | -0.7 | (1.2) | 4 | (1.2) | -1.6 | 4.3 | 0.9 | -0.3 |
| D088 | 106.12 | 39.03 | -0.2 | (1.2) | 3.5 | (1.2) | -0.5 | 4.1 | 0.3 | -0.6 |
| D089 | 106.48 | 39.07 | -1.2 | (1.2) | 4.1 | (1.2) | -2.0 | 5.1 | 0.8 | -1.0 |
| D093 | 106.56 | 38.55 | -2.2 | (1.2) | 4.3 | (1.2) | -1.6 | 4.5 | -0.6 | -0.2 |
| D095 | 105.70 | 38.44 | 0.2 | (1.2) | 4.2 | (1.2) | 0.5 | 3.7 | -0.3 | 0.5 |
| D098 | 106.68 | 37.45 | -1.2 | (1.2) | 4.5 | (1.2) | -1.4 | 4.9 | 0.2 | -0.4 |
| G001 | 105.13 | 38.05 | 0.9 | (1.2) | 3.9 | (1.2) | 0.5 | 3.9 | 0.4 | 0.0 |
| G002 | 105.89 | 37.54 | -0.3 | (1.2) | 4.2 | (1.2) | 0.5 | 4.3 | -0.8 | -0.1 |
| G003 | 105.55 | 37.52 | 0.5 | (1.2) | 4.2 | (1.2) | 0.5 | 4.3 | 0.0 | -0.1 |
| G004 | 105.18 | 37.58 | 0.1 | (1.2) | 4.6 | (1.2) | 0.4 | 4.3 | -0.3 | 0.3 |
| G005 | 105.67 | 37.36 | 0 | (1.2) | 5.1 | (1.2) | 0.5 | 4.5 | -0.5 | 0.6 |
| G006 | 105.22 | 37.24 | 0.3 | (1.2) | 5.5 | (1.2) | 0.2 | 5.3 | 0.1 | 0.2 |
| G007 | 105.88 | 36.97 | 0.5 | (1.2) | 5.2 | (1.2) | 0.3 | 5.7 | 0.2 | -0.5 |
| G008 | 105.27 | 36.93 | -0.3 | (1.2) | 5.4 | (1.2) | -0.9 | 7.2 | 0.6 | -1.8 |
| G009 | 106.36 | 36.84 | -1.5 | (1.2) | 4.8 | (1.2) | -0.2 | 5.4 | -1.3 | -0.6 |
| G011 | 105.25 | 36.66 | -1.1 | (1.2) | 7.7 | (1.2) | -1.2 | 7.9 | 0.1 | -0.2 |
| G012 | 105.62 | 36.51 | -0.3 | (1.2) | 6.9 | (1.2) | -1.5 | 7.8 | 1.2 | -0.9 |
| G013 | 105.99 | 36.28 | -1.3 | (1.2) | 6.6 | (1.2) | -1.8 | 7.8 | 0.5 | -1.2 |
| G014 | 105.29 | 36.50 | -1.7 | (1.2) | 9 | (1.2) | -1.2 | 7.9 | -0.5 | 1.1 |
| G015 | 105.62 | 36.28 | -2.7 | (1.2) | 8.4 | (1.2) | -1.5 | 7.8 | -1.2 | 0.6 |
| G016 | 106.65 | 36.10 | -2.6 | (1.2) | 5.4 | (1.2) | -1.9 | 6.9 | -0.7 | -1.5 |
| G017 | 106.22 | 36.02 | -2.8 | (1.2) | 6.4 | (1.2) | -1.9 | 8.1 | -0.9 | -1.7 |
| G018 | 105.80 | 35.96 | -3 | (1.2) | 7.5 | (1.2) | -1.7 | 7.5 | -1.3 | 0.0 |
| G019 | 104.97 | 36.09 | -1.3 | (1.2) | 7.9 | (1.2) | -1.0 | 7.7 | -0.3 | 0.2 |
| G020 | 106.20 | 35.67 | -2.7 | (1.3) | 5.7 | (1.3) | -2.0 | 7.5 | -0.7 | -1.8 |
| G023 | 105.50 | 35.61 | -1.6 | (1.3) | 6.8 | (1.3) | -1.5 | 7.2 | -0.1 | -0.4 |
| G024 | 106.58 | 35.46 | -2.5 | (1.3) | 6.1 | (1.3) | -2.6 | 8.7 | 0.1 | -2.6 |
| G025 | 106.40 | 35.46 | -1.9 | (1.3) | 6.2 | (1.3) | -1.6 | 8.1 | -0.3 | -1.9 |
| G026 | 105.02 | 35.41 | -0.7 | (1.3) | 7.8 | (1.3) | -1.0 | 7.1 | 0.3 | 0.7 |
| G027 | 106.53 | 35.24 | -2.2 | (1.3) | 6.5 | (1.3) | -2.5 | 6.3 | 0.3 | 0.2 |
| G028 | 106.01 | 35.17 | -1.7 | (1.3) | 6.6 | (1.3) | -2.1 | 6.8 | 0.4 | -0.2 |
| G030 | 105.79 | 35.08 | -2 | (1.3) | 6.8 | (1.3) | -1.9 | 6.8 | -0.1 | 0.0 |
| G031 | 106.21 | 35.01 | -3.1 | (1.3) | 7.6 | (1.3) | -2.6 | 6.7 | -0.5 | 0.9 |
| G032 | 106.82 | 34.89 | -1.7 | (1.3) | 6.5 | (1.3) | -2.5 | 5.9 | 0.8 | 0.6 |
| G033 | 105.66 | 34.87 | -1.7 | (1.3) | 6.3 | (1.3) | -1.8 | 6.7 | 0.1 | -0.4 |
| G034 | 106.16 | 34.75 | -2.4 | (1.3) | 6.5 | (1.3) | -2.1 | 6.3 | -0.3 | 0.2 |
| G035 | 105.37 | 34.79 | -1.2 | (1.3) | 6.9 | (1.3) | -1.4 | 6.6 | 0.2 | 0.3 |
| G036 | 106.40 | 34.52 | -2.7 | (1.3) | 6.5 | (1.3) | -2.3 | 6.6 | -0.4 | -0.1 |
| G037 | 105.70 | 34.59 | -1.7 | (1.3) | 7 | (1.3) | -1.8 | 6.5 | 0.1 | 0.5 |
| G038 | 107.14 | 34.43 | -1.7 | (1.3) | 6.3 | (1.3) | -2.4 | 6.6 | 0.7 | -0.3 |
| G039 | 105.81 | 34.25 | -1.8 | (1.3) | 6.7 | (1.3) | -1.6 | 7.1 | -0.2 | -0.4 |
| G042 | 104.51 | 40.74 | 0.8 | (1.2) | 3.1 | (1.2) | 0.4 | 3.2 | 0.4 | -0.1 |
| G043 | 104.81 | 40.16 | 0.9 | (1.2) | 3.4 | (1.2) | 0.4 | 3.4 | 0.5 | 0.0 |
| G077 | 104.63 | 36.91 | 0.8 | (1.2) | 8.8 | (1.2) | -0.6 | 8.1 | 1.4 | 0.7 |
| G078 | 104.13 | 36.85 | 0.6 | (1.2) | 10.7 | (1.2) | -0.2 | 8.4 | 0.8 | 2.3 |
| G085 | 104.74 | 36.43 | -1.3 | (1.2) | 9.7 | (1.2) | -0.8 | 8.0 | -0.5 | 1.7 |
| G086 | 104.36 | 36.66 | -0.3 | (1.2) | 9.9 | (1.2) | -0.4 | 8.2 | 0.1 | 1.7 |
| G107 | 104.55 | 35.54 | 0.2 | (1.2) | 8.3 | (1.2) | -0.6 | 7.2 | 0.8 | 1.1 |
| G110 | 106.68 | 34.95 | -1.9 | (1.3) | 6.6 | (1.3) | -2.5 | 5.8 | 0.6 | 0.8 |
| G114 | 104.10 | 35.04 | 0.2 | (1.3) | 8.5 | (1.3) | -0.2 | 6.8 | 0.4 | 1.7 |
| G116 | 104.54 | 35.05 | 0.4 | (1.3) | 8.6 | (1.3) | -0.6 | 6.8 | 1.0 | 1.8 |
| G120 | 104.94 | 34.71 | -1.3 | (1.3) | 7.4 | (1.3) | -0.9 | 6.5 | -0.4 | 0.9 |
| G121 | 104.92 | 34.47 | -0.6 | (1.3) | 7.7 | (1.3) | -0.9 | 6.5 | 0.3 | 1.2 |
| H003 | 105.31 | 34.11 | -1.1 | (1.3) | 6.9 | (1.3) | -1.5 | 7.5 | 0.4 | -0.6 |
| H014 | 104.07 | 34.40 | 0 | (1.3) | 8.5 | (1.3) | -0.4 | 7.1 | 0.4 | 1.4 |
| H016 | 104.38 | 34.05 | 0.3 | (1.3) | 8.6 | (1.3) | -0.3 | 8.3 | 0.6 | 0.3 |
| JB01 | 113.32 | 38.75 | -1.9 | (1.2) | 4.2 | (1.2) | -2.0 | 4.2 | 0.1 | 0.0 |
| JB05 | 113.18 | 36.23 | -1.5 | (1.2) | 4.5 | (1.2) | -2.3 | 4.8 | 0.8 | -0.3 |
| JB07 | 111.03 | 38.49 | -1.9 | (1.1) | 4.7 | (1.1) | -1.5 | 4.4 | -0.4 | 0.3 |
| JB08 | 108.09 | 35.06 | -2.2 | (1.2) | 5.4 | (1.2) | -1.9 | 5.6 | -0.3 | -0.2 |
| JB09 | 105.67 | 38.81 | -0.3 | (1.1) | 4.3 | (1.1) | 0.3 | 3.7 | -0.6 | 0.6 |
| JB27 | 105.38 | 35.14 | -1.3 | (1.2) | 6.7 | (1.2) | -1.4 | 6.8 | 0.1 | -0.1 |
| YANC | 107.44 | 37.78 | -2.5 | (1.1) | 4.6 | (1.1) | -1.9 | 4.8 | -0.6 | -0.2 |
| XIAN | 109.22 | 34.37 | -3.2 | (1.0) | 5.9 | (1.0) | -2.5 | 6.4 | -0.7 | -0.5 |

^a^ the number in the parentheses is the observation error of North component

^b^ the number in the parentheses is the observation error of East component


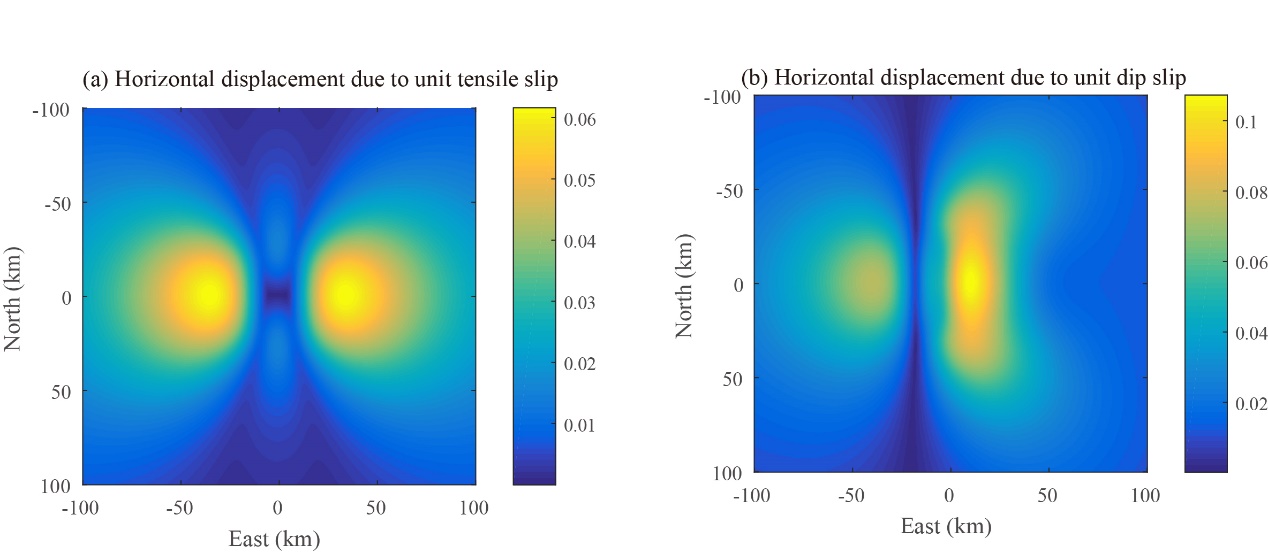


Fig. S1. Surface horizontal deformation due to a vertical fault and an inclined fault. The fault is 50km long and 30km wide, the depth of its centroid is 30km. and the strike of this fault is 0$^{\circ}$. (a) Horizontal deformation caused by 0.5 m tensile slip of a vertical fault. (b) Horizontal deformation caused by 1 m dip slip of a 60$^{\circ}$ dip angle fault.

Fig. S2 Relationship between earthquake occurrence and time of each fault. The earthquake magnitude is represented by the length of each line. The red dotted line is the time after when the earthquakes above magnitude 6 is complete.


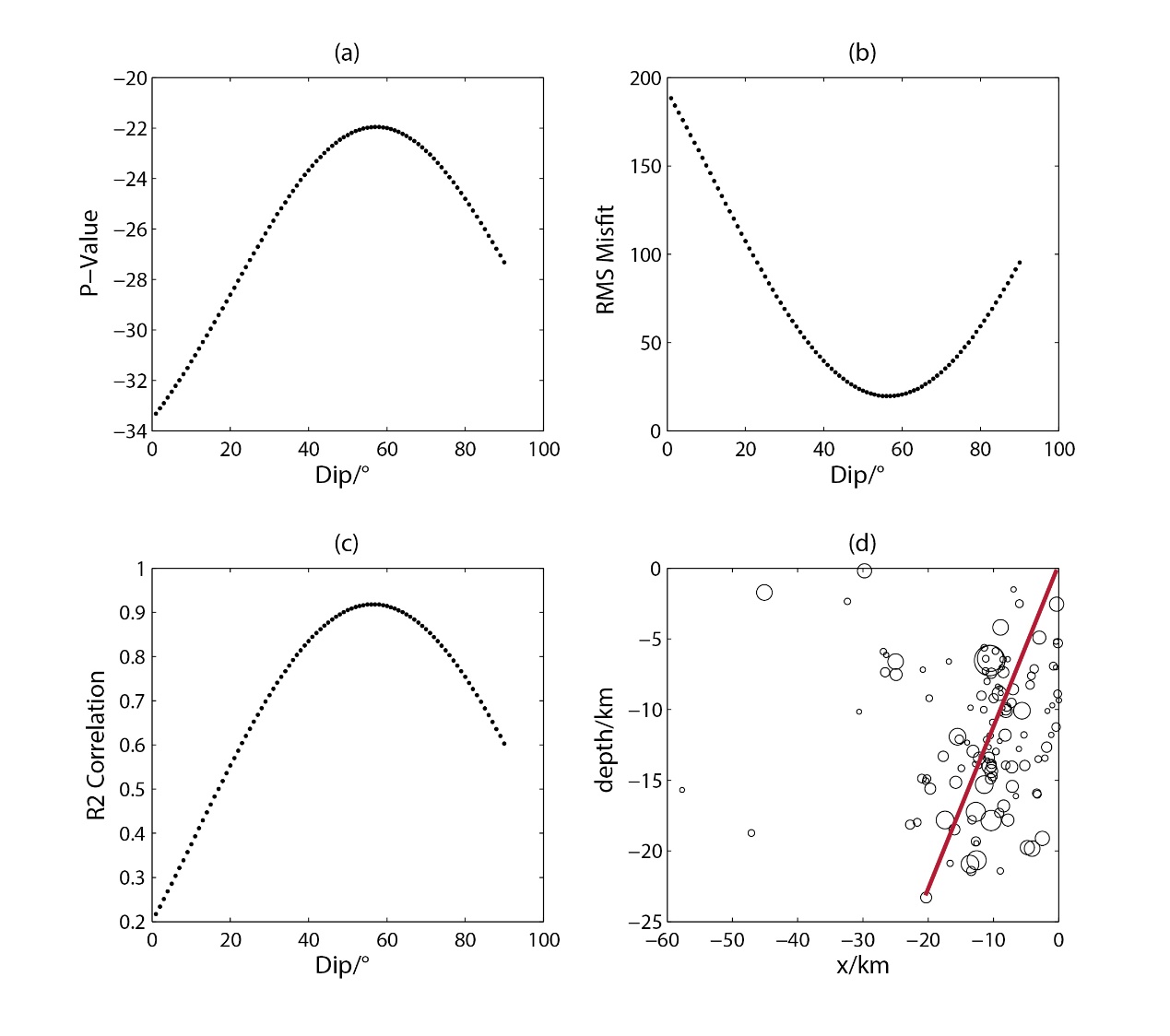


Fig. S3 Estimation of the fault’s dip angle taking Taigu Fault as an example. (a) P value versus dip angle. (b) Residual of mean square versus dip angle (c) Goodness of fit versus dip angle. (d) Red line and circles respectively represent the fault and the micro-earthquake.

Fig. S4 Locked Depths of five fault systems.
